# A Robust Method to Detect BeiDou Navigation Satellite System Orbit Maneuvering/Anomalies and Its Applications to Precise Orbit Determination

**DOI:** 10.3390/s17051129

**Published:** 2017-05-16

**Authors:** Fei Ye, Yunbin Yuan, Bingfeng Tan, Jikun Ou

**Affiliations:** 1 State Key Laboratory of Geodesy and Earth’s Dynamics, Institute of Geodesy and Geophysics, 340 Xudong Rd., Wuhan 430077, China; yybgps@whigg.ac.cn (Y.Y.); bingfengtan@whigg.ac.cn (B.T.); ojk@asch.whigg.ac.cn (J.O.); 2 University of Chinese Academy of Sciences, No. 19A Yuquan Road, Beijing 100049, China

**Keywords:** BDS, detection of anomalies and maneuvering, robust threshold, broadcast ephemeris, precise orbit determination (POD)

## Abstract

The failure to detect anomalies and maneuvering of the orbits of navigation satellite sensors will deteriorate the performance of positioning and orbit determination. Motivated by the influence of the frequent maneuvering of BDS GEO and IGSO satellites, this paper analyzes the limitations of existing methods, where BDS orbit maneuvering and anomalies can be detected, and develops a method to solve this problem based on the RMS model of orbit mutual differences proposed in this paper. The performance of this method was assessed by comparison with the health flag of broadcast ephemeris, precise orbit products of GFZ, the O-C values of a GNSS station and a conventional method. The results show that the performance of the method developed in this paper is better than that of the conventional method when the periodicity and trend items are obvious. Meanwhile, three additional verification results show that the method developed in this paper can find error information in the merged broadcast ephemeris provided by iGMAS. Furthermore, from the testing results, it can be seen that the detection of anomaly and maneuvering items do not affect each other based on the robust thresholds constructed in this paper. In addition, the precise orbit of the maneuvering satellites can be determined under the circumstances that the maneuver information detected in this paper is used, and the root mean square (RMS) of orbit overlap comparison for GEO-03/IGSO-03 in Radial, Along, Cross, 1D-RMS are 0.7614/0.4460 m, 1.8901/0.3687 m, 0.3392/0.2069 m, 2.0657/0.6145 m, respectively.

## 1. Introduction

On 31 March 2015, the first New-Generation navigation satellite of BeiDou navigation satellite system (BDS) successfully launched into orbit, which marked BDS taking a key step from the service of the Asia-Pacific area to the global network. By 2020, there will be 35 BeiDou satellites in outer space, and the system will provide worldwide users with high accuracy navigation, positioning and timing services [1,2,3,4,5]. As an important part of the system, the Geostationary Orbit (GEO) and Inclined Geosynchronous Orbit (IGSO) satellites need to be frequently maneuvered to maintain geosynchronous characteristics. Due to the impacts of various perturbations while running the orbit, satellites may be extremely disturbed, which makes the abnormal condition of satellite position occur [6,7,8]. To adjust the strategies of positioning and orbit determination in a correct and timely manner, the abnormal condition and maneuvers must be found as soon as possible after they occur. Otherwise, it will cause serious impacts on the service performance of the positioning and orbit determination [9,10,11,12,13].

The study of monitoring the orbits of space objects has long been conducted [14,15,16]; however, few studies focus on detecting the maneuvering and anomalies of navigational satellites, including GEO and IGSO satellites. At present, it is mainly based on using tracking data of orbit determination to detect the maneuvering and anomalies of GEO [17]. In general, previous research can be divided into four categories. First, radar data is used to analyze the tracking of space targets. It uses spaceborne laser/ground radar to measure the state of motion of space targets and then track these spacecrafts. An advantage is the high accuracy of measurement, but currently, the data from spaceborne laser/ground radar that can be used for navigation satellites is limited. Therefore, it is not conducive to the daily detection of the anomalies and maneuvers of navigation satellites. Second, it uses the wavelet analysis method to identify anomalies [18]; during such studies, a specific frequency signal is extracted by wavelet decomposition, after which the orbital anomalies can be distinguished using the detection theory of signal singularity. However, it is difficult to determine the wavelet basis and decomposition scale adaptively; thus, it is not easy to adaptively distinguish orbital anomalies. Third, the tracking data of orbit determination is used to monitor the maneuvering and anomalies of GEO [17]. It is based on highly accurate satellite two-way time comparison that can effectively detect orbital maneuvering, but it is too expensive to get tracking data of orbit determination for the BDS navigation satellite. It has proven difficult to popularize the application, so a method is needed that can cut costs to detect maneuvering and anomalies. Fourth, the satellite orbit calculated by the ephemeris is used to detect maneuvering and anomalies according to the jump in satellite position [19]. Raw data is easily accessible, but such research is still in its infancy, and few documents propose the orbit mutual difference RMS model of BDS broadcast ephemeris. The thresholds that can detect BDS maneuvering and anomalies do not have the robust characteristics as well. Therefore, the current body of research has its limitations in the detection of BDS anomalies and maneuvers.

This paper analyzes in depth the fourth type of research described above and attempts to utilize the BDS merged broadcast ephemeris provided by iGMAS to build an RMS model of orbit mutual difference. Furthermore, this paper proposes a robust method to detect BDS orbit anomalies and maneuvering that is sufficiently convenient to popularize the application. To verify the reliability of the established model and the validity of the proposed method according to the characteristics of BDS maneuvering and anomalies, solutions will be used to test the ability of the method in detecting maneuvering and anomalies. In addition, a third set of program will be used to analyze the impact of the maneuvering information detected in this paper on the precise orbit determination of BeiDou satellites.

## 2. A Robust Method and Its Implementation Scheme of Detection of BDS Orbit Anomalies and Maneuvering

BDS broadcast ephemeris can reflect information about satellite orbit at a fast update frequency (1 h). In this paper, BDS broadcast ephemeris was selected as the original experimental data and a method was proposed to detect BDS maneuvering and anomalies. It can be summarized as follows: use satellite orbit coordinates predicted from different epochs to the same reference epoch to calculate the RMS value of orbit mutual difference; get values of epoch-differences from the selected processing array; select robust thresholds and construct criteria according to the assumed characteristics of the RMS model of orbit mutual difference; and finally, detect orbit maneuvering and anomalies.

### 2.1. Introduction to the RMS Model of Orbit Mutual Difference

#### 2.1.1. Analysis of the RMS Values of Orbit Mutual Difference

Before introducing the RMS model of orbit mutual difference, it is necessary to analyze the RMS values of orbit mutual difference in detail, and then summarize the model according to their characteristics. Assuming that the orbital coordinates are $\left(X_{ij},Y_{ij},Z_{ij}\right)$ at the epoch $t_{j}$ which are predicted from the broadcast ephemeris at epoch $t_{i}$, and the RMS values of orbit mutual difference can be calculated from Equation (1):

(1) $$RMS_{PRN}\left(t_{i},t_{j}\right)=\sqrt{\frac{\sum_{k=1}^{k=n}{\left(X_{ik}-X_{jk}\right)}^{2}+\sum_{k=1}^{k=n}{\left(Y_{ik}-Y_{jk}\right)}^{2}+\sum_{k=1}^{k=n}{\left(Z_{ik}-Z_{jk}\right)}^{2}}{3n}}$$

where, PRN is the ID of a satellite, and n is the number of epochs contained in the broadcast ephemeris which is needed to be analyzed. 

Considering the symmetry of $RMS_{PRN}$, for simplicity, it can be discussed later, then we define the variable $rms_{PRN}(t)$ at the epoch t according to Equation (2):

(2) $$\begin{array}{c} rms_{PRN}=\left[\begin{array}{cccc} RMS_{PRN}(t_{ref},t_{1}) & RMS_{PRN}(t_{ref},t_{2}) & \ldots & RMS_{PRN}(t_{ref},t_{n}) \end{array}\right]\\ rms_{PRN}(t)=RMS_{PRN}(t_{ref},t) \end{array}\}$$

where, making $rms_{PRN}$ as the processing array, $t_{ref}$ is the selected reference epoch, $t=t_{1},t_{2},t_{3},\ldots ,t_{n}$, and this paper is based on the variable $rms_{PRN}(t)$ to start the discussion and research.

The difference operator ∇ [20] is defined as follows:

(3) $$\begin{array}{c} \nabla rms_{PRN}(t_{m})=rms_{PRN}(t_{m})-rms_{PRN}(t_{m-1})\\ \nabla^{k}rms_{PRN}(t_{m})=\nabla (\nabla^{k-1}rms_{PRN}(t_{m})) \end{array}\}$$

**Figure 1:**
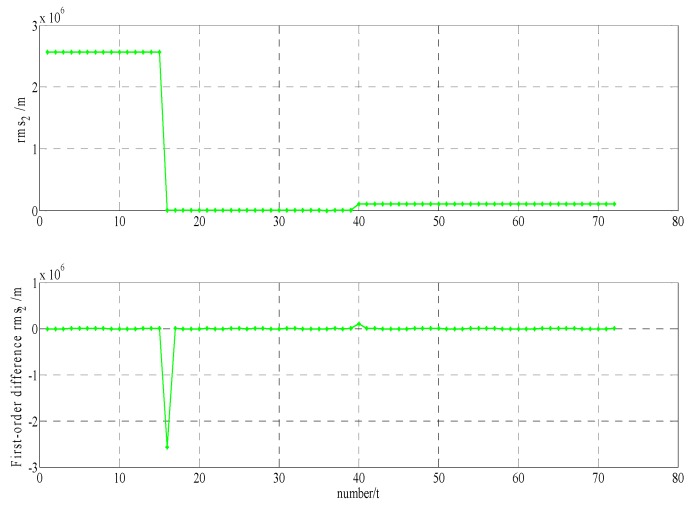
The results for $rms_{2}(t)$ and and $\nabla rms_{2}(t)$.

**Figure 2:**
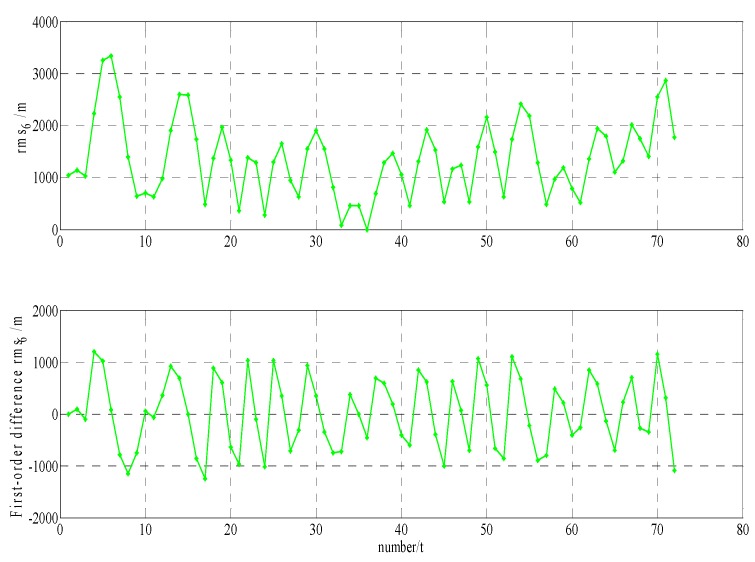
The results for $rms_{6}(t)$ and $\nabla rms_{6}(t)$.

**Figure 3:**
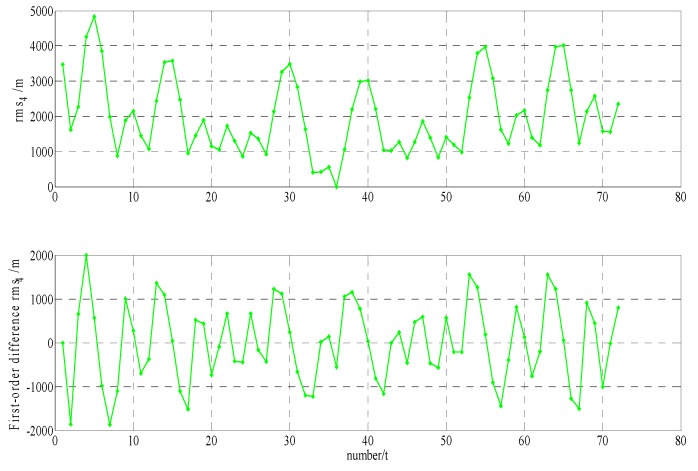
The results for $rms_{4}(t)$ and $\nabla rms_{4}(t)$.

**Figure 4:**
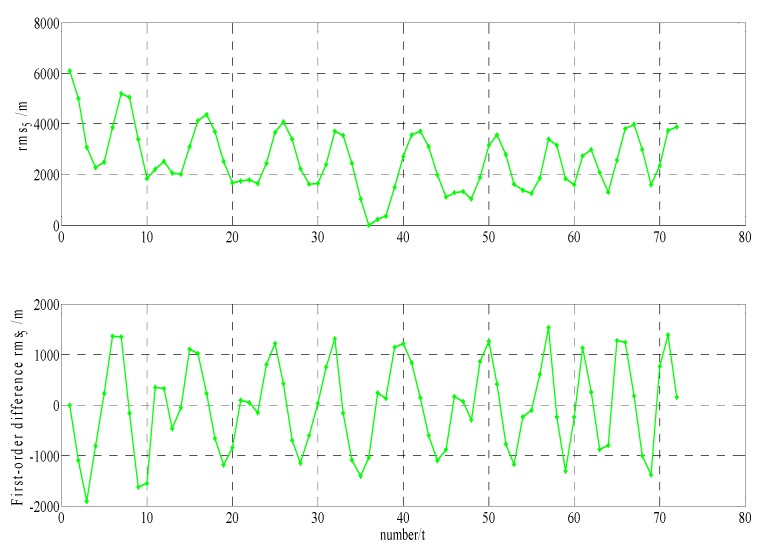
The results for $rms_{5}(t)$ and $\nabla rms_{5}(t)$.

**Figure 5:**
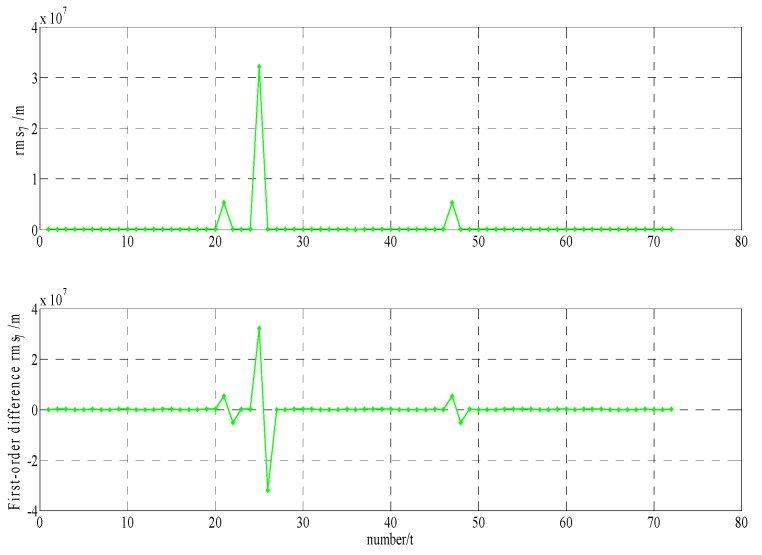
The results for $rms_{7}(t)$ and $\nabla rms_{7}(t)$.

**Figure 6:**
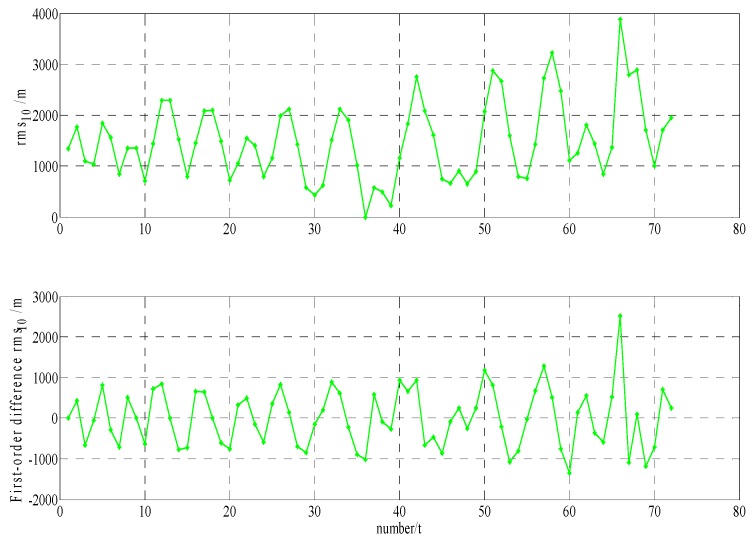
The results for $rms_{10}(t)$ and $\nabla rms_{10}(t)$.

**Figure 7:**
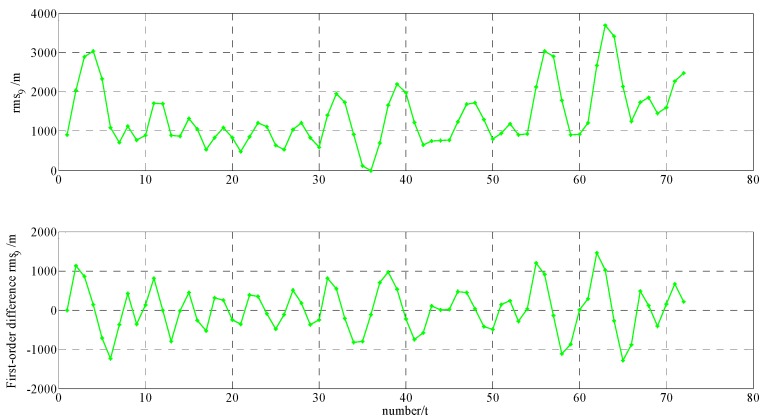
The results for $rms_{9}(t)$ and $\nabla rms_{9}(t)$.

In order to build a model that conforms to the characteristics of the variable $rms_{PRN}(t)$, a large number of experiments were analyzed, but only some of them are displayed here(the data is 27 June 2016–11 July 2016, each analysis period includes a three-day broadcast ephemeris, that is, n=72), and the others are very similar. Figure 1 and Figure 2 shows the results for $rms_{2}(t)$/$rms_{6}(t)$ and $\nabla rms_{2}(t)$/$\nabla rms_{6}(t)$ at 27 June 2016–29 June 2016; Figure 3 and Figure 4 shows the results for $rms_{4}(t)$/$rms_{5}(t)$ and $\nabla rms_{4}(t)$/$\nabla rms_{5}(t)$ at 30 June 2016–2 July 2016; Figure 5 and Figure 6 shows the results for $rms_{7}(t)$/$rms_{10}(t)$ and $\nabla rms_{7}(t)$/$\nabla rms_{10}(t)$ at 3 July 2016–5 July 2016; Figure 7 shows the results for $rms_{9}(t)$ and $\nabla rms_{9}(t)$ at 9 July 2016–11 July 2016 (Section 3 will test PRN 1/3/8, so they are not displayed here). 

The upper part of each figure represents $rms_{PRN}(t)$, and the lower part of each figure represents $\nabla rms_{PRN}(t)$ (that is the First-order difference of $rms_{PRN}(t)$).

It can be seen from the above figures that the change in $rms_{PRN}(t)$ over time shows a periodicity and trend characteristics obviously. Figure 1 and Figure 5 showed that the orbital anomaly only affects $rms_{PRN}(t)$ at the current epoch, but the orbital maneuver has an influence on $rms_{PRN}(t)$ at the current epoch and the subsequent epoch, respectively. After the first-order difference, the effects of periodicity and trend can be removed, and when there is no orbital anomaly and maneuver, $\nabla rms_{PRN}(t)$ will be displayed as the ergodic Gaussian random process.

#### 2.1.2. Determination on the RMS Model of Orbit Mutual Difference

Based on above analysis, the RMS value of the BDS orbit mutual difference consists of trend items ($TR_{PRN}$), periodicity items ($P_{PRN}$), orbit anomaly items ($\delta rms_{PRN}$), orbit maneuver items ($Crms_{PRN}$) and gauss white noise (ε). Then, we believe that the RMS value of orbit mutual difference meets the model at any epoch t  ( $t=t_{1},t_{2},t_{3},\ldots ,t_{n}$):

(4) $$rms_{PRN}\left(t\right)=TR_{PRN}\left(t\right)+P_{PRN}\left(t\right)+\delta rms_{PRN}\left(t\right)+Crms_{PRN}\left(t\right)+\varepsilon \left(t\right)$$

where, E(ε)=0 (E stands for mathematical expectation), but also $\delta rms_{PRN}$, $Crms_{PRN}$ and ε are independent to each other, that is:

(5) $$\begin{array}{c} E(\delta rms_{PRN}(t),Crms_{PRN}(t))=0\\ E(\delta rms_{PRN}(t),\varepsilon (t))=0\\ E(Crms_{PRN}(t),\varepsilon (t))=0 \end{array}\}$$

From Section 2.1.1 and Equation (3), we can see that the effects of periodicity and trend can be removed after the first-order difference, that is:

(6) $$\begin{array}{c} \begin{array}{ll} \nabla rms_{PRN}\left(t\right) & =\nabla TR_{PRN}\left(t\right)+\nabla P_{PRN}\left(t\right)+\nabla \delta rms_{PRN}\left(t\right)+\nabla Crms_{PRN}\left(t\right)+\nabla \varepsilon \left(t\right)\\ & \approx \nabla \delta rms_{PRN}\left(t\right)+\nabla Crms_{PRN}\left(t\right)+\nabla \varepsilon \left(t\right) \end{array}\\ E(\nabla rms_{PRN}(t))=E(\nabla \delta rms_{PRN}\left(t\right))+E(\nabla Crms_{PRN}\left(t\right))+E(\nabla \varepsilon \left(t\right)) \end{array}\}$$

According to the invariance property of linear transformations of Gaussian distributions, if maneuvering and anomalies do not occur in the satellite orbit, $\nabla rms_{PRN}(t)$ is a Gaussian random variable with zero expectation value as well:

(7) $$E(\nabla rms_{PRN}(t))=E(\nabla \delta rms_{PRN}\left(t\right))+E(\nabla Crms_{PRN}\left(t\right))+E(\nabla \varepsilon \left(t\right))=E(\nabla \varepsilon \left(t\right))=0$$

In contrast, $\nabla rms_{PRN}(t)$ will destruct Gaussian distributions, and based on this, a new method to detect maneuvering and anomalies is proposed.

### 2.2. A Robust Method of Detection of Orbit Anomalies and Maneuvering for BDS Satellite

Supposing the orbital arc (size of the sliding ephemeris window) of a satellite contains data of n epochs, and the epoch $t_{m}\left(t_{m}>1\right)$ is detected, then the steps for the detection of orbit anomalies and maneuvering for a BDS satellite are as follows:

(1) Select n as the size of the sliding ephemeris window.
(2) Use the corresponding data of the broadcast ephemeris to calculate the orbital coordinate $\left(X_{ij},Y_{ij},Z_{ij}\right)$ at the epoch $t_{j}$ which are predicted from the broadcast ephemeris at epoch $t_{i}$ (where i,j=1,2,3,…,n).
(3) Calculate the $RMS_{PRN}\left(i,j\right)$ value of orbit mutual difference according to Equation (1).
(4) Select the reference epoch $t_{ref}$ and get the values of the variable $rms_{PRN}(t)$ according to Equation (2).
(5) The first-order difference operation is used for $rms_{\mathrm{PRN}}(t)$ according to Equation (3), and making $\nabla rms_{PRN}\left(t_{1}\right)=0$:
  ① When satellite maneuvering occurs at the epoch $t_{m}$, the $rms_{PRN}(t)$ value is calculated as follows:
    (8) $$\begin{array}{c} rms_{PRN}\left(t_{a}\right)=\varepsilon \left(t_{a}\right)\\ rms_{PRN}\left(t_{m}\right)=Crms_{PRN}\left(t_{m}\right)+\varepsilon \left(t_{m}\right)\\ rms_{PRN}\left(t_{b}\right)=Crms_{PRN}\left(t_{m}\right)+\varepsilon \left(t_{b}\right) \end{array}\}$$
    where:
    (9) $$\begin{array}{ccc} \underset{t_{a}}{\underset{︸}{\begin{array}{cccc} t_{1} & t_{2} & \ldots & t_{m-1} \end{array}}} & t_{m} & \underset{t_{b}}{\underset{︸}{\begin{array}{cccc} t_{m+1} & t_{m+2} & \ldots & t_{n} \end{array}}} \end{array}$$
    after the first-order difference operation, $\nabla rms_{PRN}(t)$ can obtain:
    (10) $$\begin{array}{c} \begin{array}{c} \nabla rms_{PRN}(t_{a})=\nabla \varepsilon \left(t_{a}\right)\\ \nabla rms_{PRN}\left(t_{m}\right)=Crms_{PRN}\left(t_{m}\right)+\nabla \varepsilon \left(t_{m}\right) \end{array}\\ \nabla rms_{PRN}(t_{b})=\nabla \varepsilon \left(t_{b}\right) \end{array}\}$$
    (11) $$\begin{array}{c} \begin{array}{c} E\left(\nabla rms_{PRN}(t_{a})\right)=E\left(\nabla \varepsilon \left(t_{a}\right)\right)=0\\ E\left(\nabla rms_{PRN}\left(t_{m}\right)\right)=E\left(Crms_{PRN}\left(t_{m}\right)\right)+E\left(\nabla \varepsilon \left(t_{m}\right)\right)=E\left(Crms_{PRN}\left(\left|e-r\right|\right)\right) \end{array}\\ E\left(\nabla rms_{PRN}(t_{b})\right)=E\left(\nabla \varepsilon \left(t_{b}\right)\right)=0 \end{array}\}$$
  ② When satellite anomalies occur at the epoch $t_{m}$, the $rms_{PRN}(t)$ value is calculated as follows:
    (12) $$\begin{array}{c} rms_{PRN}\left(t_{a}\right)=\varepsilon \left(t_{a}\right)\\ rms_{PRN}\left(t_{m}\right)=\delta rms_{PRN}\left(t_{m}\right)+\varepsilon \left(t_{m}\right)\\ rms_{PRN}\left(t_{b}\right)=\varepsilon \left(t_{b}\right) \end{array}\}$$
    After a one order difference operation, $\nabla rms_{PRN}(t)$ can obtain:
    (13) $$\begin{array}{c} \nabla rms_{PRN}(t_{a})=\nabla \varepsilon \left(t_{a}\right)\\ \nabla rms_{PRN}\left(t_{m}\right)=\delta rms_{PRN}\left(t_{m}\right)+\nabla \varepsilon \left(t_{m}\right)\\ \nabla rms_{PRN}(t_{m+1})=-\delta rms_{PRN}\left(t_{m}\right)+\nabla \varepsilon \left(t_{m+1}\right)\\ \nabla rms_{PRN}(t_{c})=\nabla \varepsilon \left(t_{c}\right) \end{array}\}$$
    (14) $$\begin{array}{c} E\left(\nabla rms_{PRN}(t_{a})\right)=E\left(\nabla \varepsilon \left(t_{a}\right)\right)=0\\ E\left(\nabla rms_{PRN}\left(t_{m}\right)\right)=E\left(\delta rms_{PRN}\left(t_{m}\right)\right)+E\left(\nabla \varepsilon \left(t_{m}\right)\right)=E\left(\delta rms_{PRN}\left(\left|e-r\right|\right)\right)\\ E\left(\nabla rms_{PRN}(t_{m+1})\right)=E\left(-\delta rms_{PRN}\left(t_{m}\right)\right)+E\left(\nabla \varepsilon \left(t_{b}\right)\right)=-E\left(\delta rms_{PRN}\left(\left|e-r\right|\right)\right)\\ E\left(\nabla rms_{PRN}(t_{c})\right)=E\left(\nabla \varepsilon \left(t_{c}\right)\right)=0 \end{array}\}$$
    where:
    (15) $$\begin{array}{cccc} \underset{t_{a}}{\underset{︸}{\begin{array}{cccc} t_{1} & t_{2} & \ldots & t_{m-1} \end{array}}} & t_{m} & t_{m+1} & \underset{t_{c}}{\underset{︸}{\begin{array}{cccc} t_{m+2} & t_{m+3} & \ldots & t_{n} \end{array}}} \end{array}$$
  ③ When the satellite is within normal status at the epoch $t_{m}$, the $rms_{PRN}(t)$ value is calculated as follows:
    (16) $$\begin{array}{c} rms_{PRN}\left(t_{a}\right)=\varepsilon \left(t_{a}\right)\\ rms_{PRN}\left(t_{m}\right)=\varepsilon \left(t_{m}\right)\\ rms_{PRN}\left(t_{b}\right)=\varepsilon \left(t_{b}\right) \end{array}\}$$
    After a one order difference operation, $\nabla rms_{PRN}(t)$ can obtain:
    (17) $$\begin{array}{c} \begin{array}{c} \nabla rms_{PRN}(t_{a})=\nabla \varepsilon \left(t_{a}\right)\\ \nabla rms_{PRN}\left(t_{m}\right)=\nabla \varepsilon \left(t_{m}\right) \end{array}\\ \nabla rms_{PRN}(t_{b})=\nabla \varepsilon \left(t_{b}\right) \end{array}\}$$
    (18) $$\begin{array}{c} \begin{array}{c} E\left(\nabla rms_{PRN}(t_{a})\right)=E\left(\nabla \varepsilon \left(t_{a}\right)\right)=0\\ E\left(\nabla rms_{PRN}\left(t_{m}\right)\right)=E\left(\nabla \varepsilon \left(t_{m}\right)\right)=0 \end{array}\\ E\left(\nabla rms_{PRN}(t_{b})\right)=E\left(\nabla \varepsilon \left(t_{b}\right)\right)=0 \end{array}\}$$
(6) Because the median is provided with high robust and high breakdown contamination rates [21,22,23,24,25], this paper uses this characteristic to suppress the influence of anomalies when maneuvering is detected. Considering the characteristics of the processing array, the absolute value of the array $\nabla rms_{PRN}(t)$ is first obtained by step (5), and then, according to the relationship between median and mean square error, the array of absolute value was used to calculate robust variance factors:
  (19) $$\sigma^{0}=med\left(\mathrm{abs}(\nabla rms_{PRN}(t)\right))/0.6745$$
(7) According to past experience, the paper chooses four times (it can be appropriate to enlarge if needed) the variance factors as thresholds of detection:
  (20) $$\begin{array}{c} T_{1}=4\times \sigma^{0}\\ T_{2}=-4\times \sigma^{0} \end{array}\}$$
(8) For the detection of maneuvering and anomalies at epoch $t_{m}$, when satellite maneuvering or anomalies occur, $\nabla rms_{PRN}(t)$ will destruct Gaussian distributions; according to this principle, the detection criteria were set as follows:
  ① When satellite maneuvering occurs at the epoch $t_{m}$, the following criteria can be obtained by Equations (8)–(11):
    (21) $$\begin{array}{c} \nabla rms_{PRN}\left(t_{m}\right)>T_{1}\\ \nabla rms_{PRN}\left(t_{m+1}\right)\geq T_{2} \end{array}\}\;or\;\begin{array}{c} \nabla rms_{PRN}\left(t_{m}\right)\leq T_{1}\\ \nabla rms_{PRN}\left(t_{m+1}\right)<T_{2} \end{array}\}$$
  ② When satellite anomalies occur at the epoch $t_{m}$, the following criteria can be obtained by Equations (12)–(15):
    (22) $$\begin{array}{c} \nabla rms_{PRN}\left(t_{m}\right)>T_{1}\\ \nabla rms_{PRN}\left(t_{m+1}\right)<T_{2} \end{array}\}\;\mathrm{or}\;\begin{array}{c} \nabla rms_{PRN}\left(t_{m}\right)<T_{2}\\ \nabla rms_{PRN}\left(t_{m+1}\right)>T_{1} \end{array}\}$$
  ③ When the satellite stays within normal status at the epoch $t_{m}$, the following criteria can be obtained by Equations (16)–(18):
    (23) $$\begin{array}{c} \nabla rms_{PRN}\left(t_{m}\right)\leq T_{1}\\ \nabla rms_{PRN}\left(t_{m+1}\right)\geq T_{2} \end{array}\}\;\mathrm{or}\;\begin{array}{c} \nabla rms_{PRN}\left(t_{m}\right)\geq T_{2}\\ \nabla rms_{PRN}\left(t_{m+1}\right)\leq T_{1} \end{array}\}$$
(9) The whole window moves backward at an epoch, repeating steps (2–8), until all epochs are finished.

The anomalies and maneuvering of a BDS satellite orbit can be detected by the above steps (1–9). The following sections will use the BDS broadcast ephemeris to validate and analyze this proposed method.

## 3. Validation and Analysis

To test the ability of the proposed method, which is used to detect maneuvering and anomalies, this paper uses the data of the merged broadcast ephemeris provided by iGMAS and utilizes the following programs to detect maneuvering and anomalies of GEO and IGSO. Considering the fact that it is not currently available to get real information on BDS orbit anomalies and maneuvering**.** So the test results of the proposed method (method one) are mainly compared with the health flag of broadcast ephemeris, precise orbit products of GFZ, and the O-C values of station BJF1/WUH1, which is obtained by that the station-satellite distance minus the observation distance, the station coordinate is provided by the observation file, and the satellite coordinate is fitted using BDS broadcast ephemeris. It is possible to verify whether or not the RMS model of orbit mutual difference is reliable. Moreover, it can also be shown whether the method is effective or not. Furthermore, in order to analyze the performance of the RMS model and the detection method proposed in this paper, the proposed method will also be compared with the conventional method (method two) using $rms_{PRN}(t)$ obtained by step (4) to directly detect maneuvering, in which the threshold of detection is the empirical value, which is equal to 5000, as proposed by [19]. In addition, in order to analyze the influence of orbital maneuver on precise orbit determination of BeiDou satellites, the validity of the maneuver information detected in this section will be analyzed in the last part of this section from the perspective of precise orbit determination.

Since the solutions of precise orbit determination often use single-day, three-day and seven-day broadcast ephemeris products as the initial orbit, the size of the windows selected for use in the test were single-day, three-day and seven-day.

Program one: The testing for the detecting ability of BDS orbit maneuvering of the robust method proposed in this paper. For GEO-03, the time is from 5 January 2015 00:00 to 7 January 2015 23:00; for IGSO-03, the time is from 9 January 2015 05:00 to 10 January 2015 05:00. The data comes from the BDS merged broadcast ephemeris provided by iGMAS.

**Figure 8:**
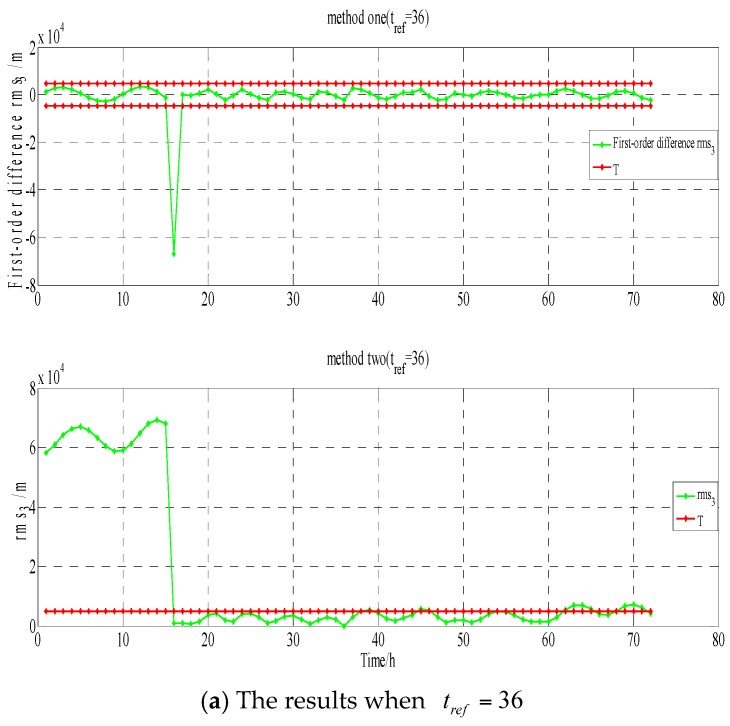

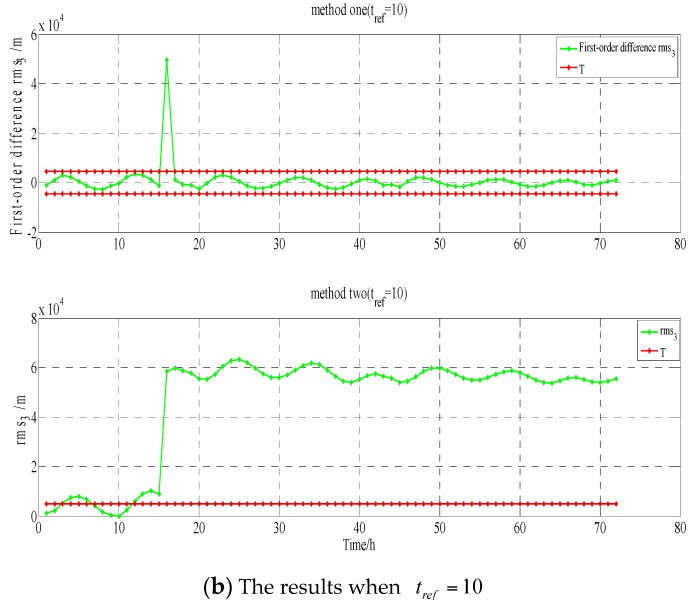
The results for method one ($\nabla rms_{3}(t)$/method two ($rms_{3}(t)$) when $t_{ref}=36$ and $t_{ref}=10$, respectively.

**Figure 9:**
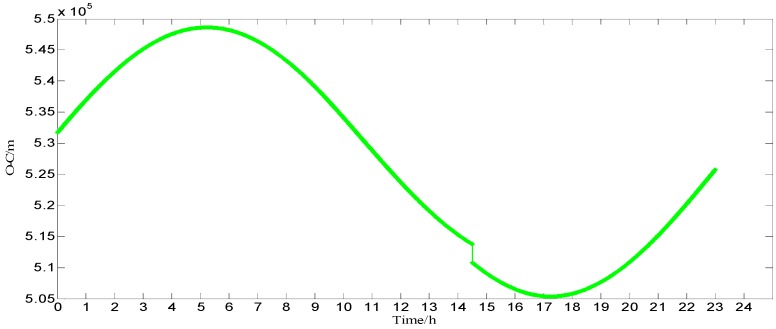
Results of O-C of GEO-03 at WUH1.

(1) Testing and analysis in GEO-03(PRN3). The testing results of methods one and two are shown in Figure 8 (X-axis represents time, unit is hours; Y-axis represents value calculated by Equation (3), unit is meters); from 5 January 2015 9:00 to 5 January 2015 14:00, the health flag of the broadcast ephemeris showed that GEO-03 is in an unhealthy state; on 5 January 2015, among the products of precise orbits, GFZ did not calculate the coordinates of GEO-03; at station WUH1, the O-C values of GEO-03 are shown in Figure 9 (X-axis represents time, unit is hours; Y-axis represents that station-satellite distance minus observed distance of L3, unit is meters);

From Figure 8 and the other three verification methods for GEO-03, the testing results from 5 January 2015, at 14:00–15:00, are shown in Table 1.

**Figure 10:**
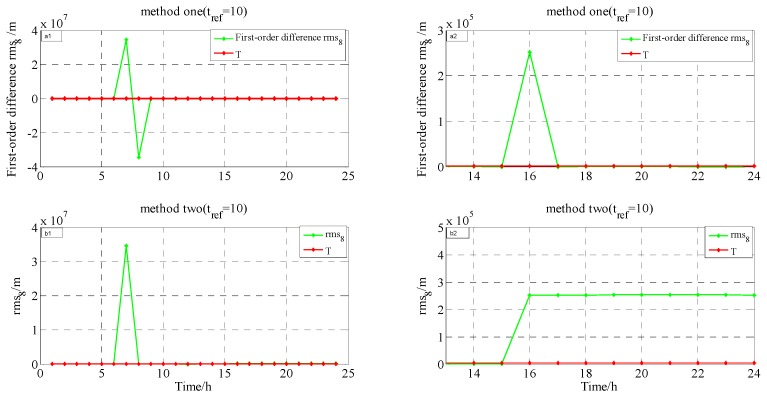
(**a1**) The results for method one ($\nabla rms_{8}(t)$), (**a2**) The results for partial enlarged (**a1),** and (**b1**) The results for method two ($rms_{8}(t)$ ), (**b2**) The results for partial enlarged (**b1**).

**Figure 11:**
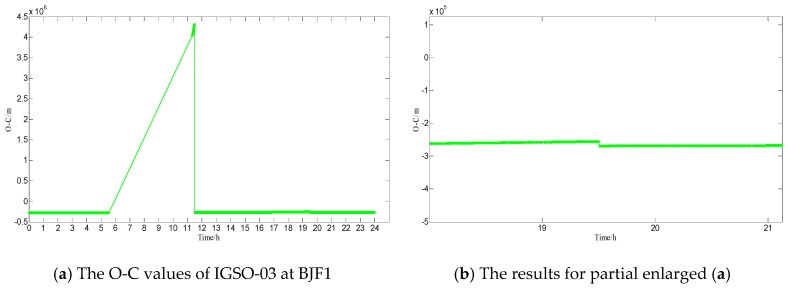
Results of O-C of IGSO-03 at BJF1 (The graph on the right is a partial enlarged view of the left).

The results in Table 1 show that on 5 January 2015, at 14:00–15:00, both methods detect that GEO-03 maneuvering may occur: the health flag of broadcast ephemeris shows that GEO-03 is in a unhealthy state, but in the previous five hours, the health flag shows that GEO-03 is also in an unhealthy state; GFZ might think that GEO-03 is in an unhealthy state; and, there is a jump in the O-C value at WUH1, which may represent that GEO-03 is in an unhealthy state. Furthermore, it can be concluded from Figure 8 that the detection result of the conventional method is affected by the selected reference epoch, and the result of the epoch with far from the reference epoch is obviously affected by the periodicity and trend term, so that the detection result is not completely correct. But the new method is not affected by these factors:

(2) Testing and analysis in IGSO-03(PRN8). The testing results of methods one and two are shown in Figure 10. From 9 January 2015 14:00 to 9 January 2015 20:00, the health flag of broadcast ephemeris showed that IGSO-03 is in an unhealthy state, while at 11:00, the health flag is in a healthy state. On 9 January 2015, among the products of precise orbits, GFZ did not calculate the coordinate of IGSO-03. At station BJF1, the O-C values of IGSO-03 are shown in Figure 11 (X-axis represents time, unit is hours; Y-axis represents that station-satellite distance minus observed distance of B1, unit is meters);

From Figure 10 and the other three verification methods for IGSO-03, from 9 January 2015, at 10:00–11:00 and 19:00–20:00, the testing results are shown in Table 2 and Table 3, respectively.

From Figure 10, it can be seen that anomalies do not affect the detection of maneuvering. From the results in Table 3, it can be seen that on 9th January 2015, at 19:00–20:00, both methods detect that IGSO-03 maneuvering may occur: the health flag of broadcast ephemeris shows that IGSO-03 is in an unhealthy state, while in the previous five hours and the later one hour, the health flag shows that IGSO-03 is also in an unhealthy state; GFZ may think that IGSO-03 is in an unhealthy state; and, there is a jump in the O-C value at BJF1, which may represent that IGSO-03 is in an unhealthy state.

Program two: Testing for the detecting ability of BDS orbit anomalies by the robust method proposed in this paper. For GEO-01, the time was from 4th January 2015 00:00 to 10th January 2015 23:00. The data comes from the BDS merged broadcast ephemeris provided by iGMAS.

**Figure 12:**
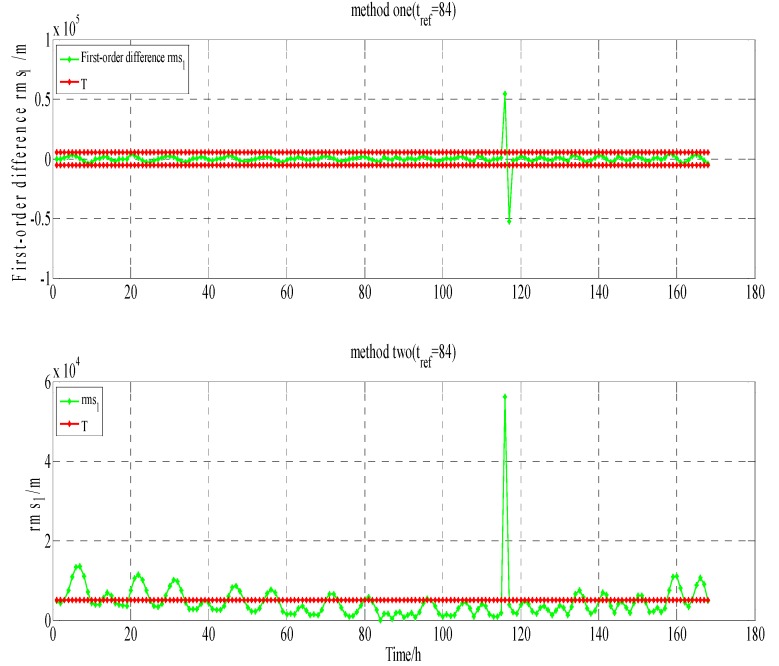
The results for method one($\nabla rms_{1}(t)$/method two($rms_{1}(t)$).

**Figure 13:**
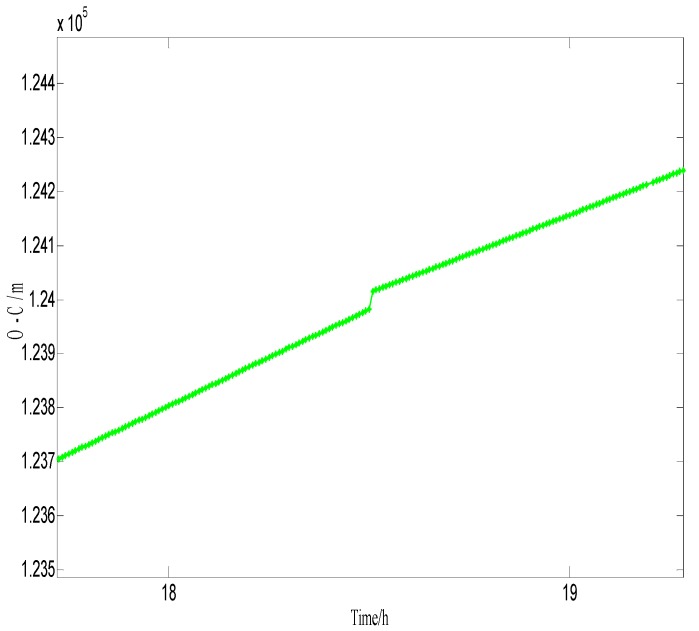
Results of O-C of GEO-01 at BJF1.

Testing and analysis in GEO-01(PRN1). The testing results of method one and two are shown in Figure 12. On 8th January 2015 19:00, the health flag of broadcast ephemeris showed that GEO-01 is in a healthy state. Among the products of precise orbit, GFZ calculated the coordinates of GEO-01. At station BJF1, the O-C values of GEO-01 are shown in Figure 13 (X-axis represents time, unit is hours; Y-axis represents that station-satellite distance minus observed distance of B1, unit is meters).

From Figure 12 and other three verification ways for GEO-01, on 8 January 2015, at 18:00–19:00, the testing results are shown in Table 4.

From the results in Table 4, it can be seen that on 8 January 2015, at 18:00–19:00, both methods detected that GEO-01 anomalies may occur that are caused by decoding errors or other reasons: the health flag of broadcast ephemeris show that GEO-01 is in a healthy state; GFZ may think that GEO-01 is in a healthy state; and, there is a jump in the O-C value at BJF1, which may represent that GEO-01 is in an unhealthy state. Moreover, it can be seen from Figure 12 that the results at both ends of the detection window due to the impact of the periodicity and trend term is clearly wrong.

From Figure 10, it can be seen that maneuvering does not affect detection of anomalies. From the results in Table 2, it can be seen that on 9 January 2015, at 10:00–11:00, both methods detect that IGSO-03 may occur anomalies that are caused by decoding errors or other reasons: the health flag of broadcast ephemeris show that IGSO-03 is in a healthy state; GFZ may think that IGSO-03 is in an unhealthy state; and, there is a jump in the O-C value at BJF1, which may represent that IGSO-03 is in an unhealthy state.

Program three: In order to further test the effect of orbital maneuvering information on the precise orbit determination, the orbit maneuvering information is used for precise orbit determination. As neither GFZ nor WHU provides the precise orbits of the two satellites when these two satellites maneuvered their orbits, orbit overlap comparison is used to analyze the precision of the orbit determination.

**Figure 14:**
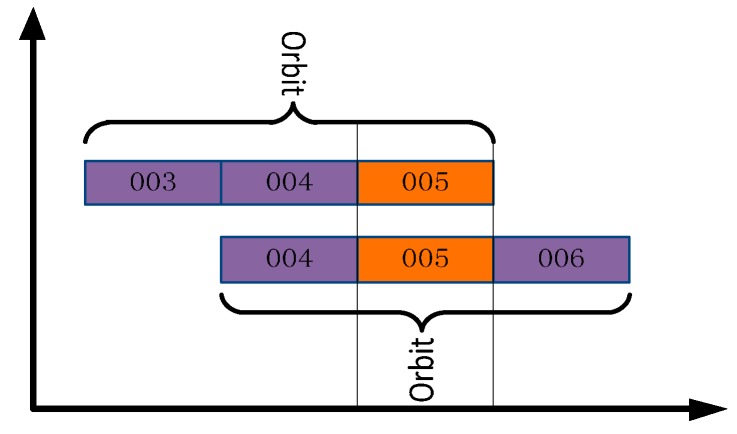
Comparison Strategy of GEO-03.

**Figure 15:**
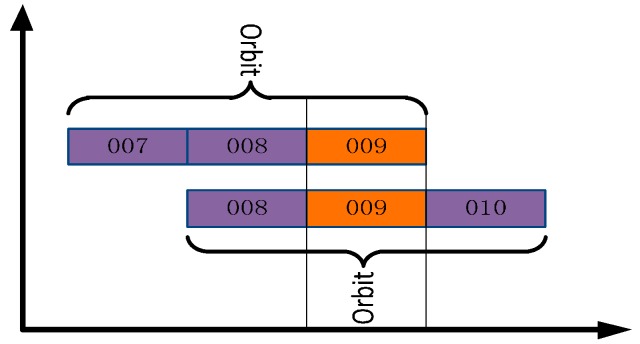
Comparison Strategy of IGSO-03.

(1) GEO-03 precise orbit maneuvering test. In this test, the BDS measured data of 20 stations from MGEX and 10 stations from iGMAS from 3 to 6 January 2015 were used to obtain the final precision orbit using three-day long arc POD. The comparison strategy is shown in Figure 14, and the statistical results of orbit overlap comparison in radial direction, along direction, cross direction, 1D-RMS are shown in Table 5;
(2) IGSO-03 precise orbit maneuvering test. In this test, the BDS measured data from 7 to 10 January 2015 were used to obtain the final precision orbit. The comparison strategy is shown in Figure 15, and the statistical results of orbit mutual difference of overlapping arc in radial direction, along direction, cross direction, 1D-RMS are shown in Table 6;

From the results of GEO-03 and IGSO-03 precise orbit maneuvering tests, we can see that it is difficult to provide precise orbit determination results for GEO-03 and IGSO-03 when there is no satellite orbit maneuvering information. However, when using the maneuver information detected in this paper, the precise orbit determination results of these two satellites not only can be determined, but also the accuracy of the orbit determination is equivalent to that of the same type of orbit determination (The accuracy of GEO/IGSO are meters and decimeters, respectively). What’s more, the GEO-03 is more accurate than the GEO-02 in terms of orbit determination accuracy, and the differences in 1D-RMS accuracy between IGSO-03 and IGSO-02, IGSO-04 1D-RMS are in the order of decimeters.

The above programs can verify that the ability of the robust method proposed in this paper, which can detect BDS orbit maneuvering and anomalies, is effective and that they can also verify the reliability of the proposed RMS model. In the process of satellite orbit determination, if the maneuvering moments detected in this paper is segmented, this satellite can perform normal precise orbit determination. The results of precision orbit determination when the maneuver information detected in this paper was used are shown that the 1D-RMS is 2.0657 m before the maneuver of GEO-03, and the 1D-RMS is 0.6145 m before the maneuver of IGSO-03. It should be said that due to the limited resolution of the maneuver detection, the maneuver time points aren’t determined precisely, so, only the orbit arcs before the maneuver time points are tested. 

## 4. Conclusions and Outlook

In this study, an RMS model of orbit mutual differences was established according to characteristics of orbital prediction accuracy, and a robust method to detect BDS orbit anomalies and maneuvering was proposed based on the RMS model. 

BDS orbit anomalies and maneuvering were detected using the proposed method in this study, and the testing results were then verified by a health flag of broadcast ephemeris, precise orbit products of GFZ, the O-C values of station BJF1/WUH1 and the conventional method. By comparison with the results of the conventional method, it can be seen that when the periodicity and trend items are obvious, the performance of the method proposed in this paper is more stable than the conventional method. Moreover, the empirical threshold used by the conventional method does not always meet the actual requirements, while the robust thresholds constructed in this paper have a higher reliability. From the other three verification results, it can be seen that the RMS model of orbit mutual difference proposed in this paper is reliable; furthermore, the detecting method of maneuvering and anomalies developed in this paper is effective. In addition, this method can also detect inaccurate information in the merged broadcast ephemeris provided by iGMAS, such as the error health flag of broadcast ephemeris. The testing results also show that, based on the robust thresholds constructed in this paper, anomalies do not affect the detection of maneuvering; meanwhile, maneuvering does not affect the detection of anomalies, and this method has greater consistency compared with the other verification results. During this experiment, it was shown that the sliding window and the reference epoch can be adjusted according to the actual demand; this also reflects that the method proposed in this paper has greater flexibility.

In addition, the maneuver information detected in this paper will have a significant impact on the precise orbit determination of satellites, which can not only realize the precise orbit determination of maneuvered satellites, but also the accuracy of the orbit determination is equivalent to that of the same type of satellite orbit determination. The experimental results show that the precise orbit determination results of maneuvered satellites can’t be obtained before using the satellite maneuver information. However, after using the maneuver information detected in this paper, the results of orbit overlap comparison of GEO-03 in radial direction, along direction, cross direction, 1D-RMS are 0.7614 m, 1.8901 m, 0.3392 m, 2.0657 m, respectively, and the results of orbit overlap comparison of IGSO-03 in radial direction, along direction, cross direction, 1D-RMS are 0.4460 m, 0.3687 m, 0.2069 m, 0.6145 m, respectively.

Although the proposed robust method to detect BDS orbit anomalies and maneuvering was successful, the results of this paper depend on BDS merged broadcast ephemeris, which is updated every hour by iGMAS. As such, the time resolution is limited, so a method to detect maneuvering and anomalies in higher time resolutions will be addressed in future work.

## Figures and Tables

**Table 1:** Results of GEO-03 maneuvering detection at 14:00–15:00.

Method One (New Method)	Method Two (Conventional Method)	The Health Flag of Broadcast Ephemeris	Precise Orbit Products of GFZ	O-C Value at WUH1
Maneuvering	Maneuvering	Unhealthy	No GEO-03	There is a jump

**Table 2:** Results of IGSO-03 maneuvering detection at 10:00–11:00.

Method One (New Method)	Method Two (Conventional Method)	The Health Flag of Broadcast Ephemeris	Precise Orbit Products of GFZ	O-C Value at BJF1
Anomalies	Anomalies	Healthy	No IGSO-03	There is a jump

**Table 3:** Results of IGSO-03 maneuvering detection at 19:00–20:00.

Method One (New Method)	Method Two (Conventional Method)	The health flag of Broadcast Ephemeris	Precise Orbit Products of GFZ	O-C Value at BJF1
Maneuvering	Maneuvering	Unhealthy	No IGSO-03	There is a jump

**Table 4:** Results of GEO-01 anomalies detection at 18:00–19:00.

Method One (New Method)	Method Two (Conventional Method)	The Health Flag of Broadcast Ephemeris	Precise Orbit Products of GFZ	O-C Value at BJF1
Anomalies	Anomalies	Healthy	Have GEO-01	There is a jump

**Table 5:** RMS of orbit overlap comparison at 5 January (unit: m).

Satellite	PRN	Radial	Along	Cross	1D-RMS
GEO-01	1	0.1389	0.2016	0.1695	0.2634
GEO-02	2	1.4875	1.7481	0.2402	2.3079
**GEO-03**	**3**	**0.7614**	**1.8901**	**0.3392**	**2.0657**
GEO-04	4	0.1435	0.3520	0.0743	0.3873
GEO-05	5	0.1576	0.4225	0.2662	0.5236
IGSO-01	6	0.0828	0.1644	0.1258	0.2230
IGSO-02	7	0.1170	0.2529	0.7386	0.7894
IGSO-03	8	0.1570	0.4986	0.4792	0.7091
IGSO-04	9	0.0787	0.0916	0.1428	0.1870
IGSO-05	10	0.0722	0.2861	0.6542	0.7177

**Table 6:** RMS of orbit overlap comparison at 9 January (unit: m).

Satellite	PRN	Radial	Along	Cross	1D-RMS
GEO-01	1	0.0554	0.0783	0.0458	0.1063
GEO-02	2	0.0686	0.1772	0.1112	0.2202
GEO-03	3	0.0940	0.1030	0.0776	0.1596
GEO-04	4	0.0288	0.1301	0.0370	0.1383
GEO-05	5	0.0565	0.0530	0.1104	0.1349
IGSO-01	6	0.0328	0.1201	0.3887	0.4082
IGSO-02	7	0.1074	0.1651	0.5064	0.5434
**IGSO-03**	**8**	**0.4460**	**0.3687**	**0.2069**	**0.6145**
IGSO-04	9	0.0775	0.1115	0.5155	0.5331
IGSO-05	10	0.0935	0.0888	0.0943	0.1598
